# *Acinetobacter baumannii*: much more than a human pathogen

**DOI:** 10.1128/aac.00801-25

**Published:** 2025-07-25

**Authors:** Santiago Castillo-Ramírez, Alejandro Aguilar-Vera, Ayush Kumar, Benjamin Evans

**Affiliations:** 1 Programa de Genómica Evolutiva, Centro de Ciencias Genómicas, Universidad Nacional Autónoma de México 61740 , Cuernavaca, Mexico; 2 Department of Microbiology, University of Manitoba 468335, Winnipeg, Manitoba, Canada; 3 Centre for Metabolic Health, Norwich Medical School, University of East Anglia 12201, Norwich, United Kingdom; 4 Centre for Microbial Interactions, Norwich Research Park 455075, Norwich, United Kingdom; Houston Methodist Hospital and Weill Cornell Medical College, Houston, Texas, USA

**Keywords:** *Acinetobacter baumannii*, antimicrobial resistance, international clones, genomic epidemiology, One Health, environment, non-human populations

## Abstract

*Acinetobacter baumannii* is a major human nosocomial pathogen. Due to this, a significant amount of knowledge has been gained about human clinical isolates over a substantial period of time. More recently, studies have begun to pay attention to non-human isolates of *A. baumannii*. In reviewing these studies, we highlight some major trends. First, *A. baumannii* has been found in a variety of sources/hosts: from diverse types of animals, to food products, to plants and even aquatic environments. Second, considering the molecular epidemiology of *A. baumannii*, two scenarios are possible. One implies transmission between human and non-human populations, and this has been described in several international clones (ICs): IC1, IC2, IC5, IC7, and IC8. In the other scenario, human populations are well differentiated from non-human populations, and there is no exchange between them. Third, in terms of antibiotic resistance in the non-human populations, these populations tend to have fewer antibiotic resistance genes, mostly intrinsic in nature. However, when non-clinical bacterial populations come into closer contact with humans, the antibiotic resistance profiles of the non-human bacterial population become more similar to those of clinical populations. Also, there are some instances of non-human isolates showing extensive drug resistance phenotypes. By far, the least studied aspect is the virulence potential of *A. baumannii* from non-human sources. A small number of studies suggest that some non-human isolates can be as virulent as the human isolates. Finally, we discuss gaps in knowledge and future research avenues when considering non-human populations of *A. baumannii* and their relationship with human populations.

## INTRODUCTION

Antimicrobial resistance is a major human health issue, and *Acinetobacter baumannii* is one of the most relevant multidrug-resistant (MDR) bacteria in hospital settings (1). This pathogen is a common cause of hospital-acquired infections worldwide. The World Health Organization has classified *A. baumannii* as the top priority pathogen for which new antibiotics are urgently required (2). There are several key factors that make *A. baumannii* a difficult-to-treat pathogen. One of these is its great capacity to acquire genes (3). Particularly relevant among the horizontally transferred genes are antibiotic resistance genes (ARGs) that can be acquired via plasmids (4, 5). Another important factor is the ability of this bacterium to survive for long periods on dry surfaces, which enhances the risk of transmission. Understandably, much of the research about this bacterium has been on aspects related to its human clinical relevance. For instance, a recent natural language processing analysis of over 5,500 research papers on *A. baumannii* has shown a significant bias toward themes related to human health (such as hospital infections, clinical treatment, and multidrug resistance) (6). That study also found that non-human infections and related themes have been considerably understudied (6). Nonetheless, in the last decade, there has been a conspicuous increase in the study of non-human populations of *A. baumannii*. This bacterium has been reported in many different animals, foods, plants, and aquatic environments (7–13). Furthermore, there is mounting evidence that *A. baumannii* can also be a veterinary pathogen (14, 15). It has even been suggested that this bacterium should be considered a One Health issue (16, 17). Knowledge of the biology and ecology of non-human populations of *A. baumannii*, or indeed for any other pathogen, is of paramount importance to better deal with the global health crisis of antibiotic resistance. Notably, antibiotic resistance is an archetypical example of a One Health problem (18), as it not only affects humans but also animals and is a worldwide issue.

In this minireview, we summarize the conceptual advances and provide a bird’s eye view of some of the non-human populations of this pathogen. The review is structured in three major themes: molecular epidemiology, antibiotic resistance, and virulence. We aim to summarize progress in those three major overarching themes. Rather than provide an exhaustive account, our goal is to distill the main trends considering these themes. In particular, we connect the findings in the non-human populations and how these relate to what is known for the major international clones (ICs) described for the human population. Importantly, we highlight the major trends structured according to the three major themes (see Table 1). To provide a coherent view, we focused on three broad non-human sources: (i) diverse types of animals (pets, livestock, and wildlife), (ii) food (meat and produce), and (iii) aquatic environments (lakes, rivers, and water waste treatment plants). We chose these as there have been sufficient studies from these sources, and thus, some patterns can be inferred. Considering animals, these go from companion animals to food-producing animals and wildlife. Aquatic environments are also diverse, from lakes and rivers to wastewater. We end the review by highlighting some conceptual advances made thus far and outlining some important challenges and opportunities for future research concerning non-human populations of *A. baumannii*.

**TABLE 1:** Major trends and unknowns of non-human *A. baumannii*

Theme	Major trends	Unknowns
Mol. Epidemiology	*A. baumannii* can be found in a wide variety of sources: animals, food, plants, and aquatic environments. Non-human isolates can form novel lineages, but they can also fall within the human ICs. Isolates from companion animals belonged to well-known human ICs (IC1, IC2, IC3, and IC7). Isolates from livestock and wildlife can be novel lineages, but also part of well-known human ICs, such as IC2 or IC8. Aquatic isolates can be novel STs, but also part of the human ICs, for instance, IC1, IC2, or IC8.	How extensive is the habitat of *A. baumannii*? Are there some lineages better equipped to inhabit different host/sources? To what extent is *A. baumannii* in food products a risk for vulnerable human patients? What makes some ICs prone to be found in different sources?
Antibiotic Resistance	Non-human populations tend to have fewer antibiotic resistance genes than human ones. Isolates from heavily human-impacted environments (for instance, wastewater) can have many antibiotic resistances. Pan- and extensively drug-resistant isolates have been described in animals.	What are the mobile genetic elements mobilizing acquired resistance in non-human populations of *A. baumannii*? How variable is the rate of exchange of antibiotic resistance genes between human and non-human populations?
Virulence	Scarce data suggest that some non-human isolates can be as virulent as some human clinical isolates.	What is the human pathogenic potential of non-human *A. baumannii*? Can *A. baumannii* be a significant pathogen to non-human species?

## MOLECULAR EPIDEMIOLOGY

### *A. baumannii* from animals

Besides humans, animals are the most well-studied hosts for *A. baumannii* (see Table 2). These go from companion animals, mainly dogs and cats, to livestock and even wildlife. However, most animal isolates have come from pets. For instance, a study analyzing 52 isolates collected from hospitalized animals, sampled between 2000 and 2008, at veterinary clinics in Germany found MDR *A. baumannii* in animals (19). These isolates were sampled mainly from cats, dogs, and horses and were assigned to IC1 and IC3 (19). Another study in 2014 reported an MDR isolate which belonged to ST2 (IC2), sampled from a cat with a urinary tract infection (UTI) (20). In another study in France, Lupo et al. found isolates also from UTIs in dogs (21). The isolates were clonally related and belonged to ST25 (Pasteur scheme), which is part of IC7. There have been few studies that have analyzed carriage in animals (22–24). For instance, a study conducted in Reunion Island, where 138 pets were sampled via mouth and rectal swabbing, found a prevalence of 6.5% and noted that most of the isolates were closely related (22). Another French study, also conducting mouth and rectal swabbing and studying *A. baumannii* carriage in pets from the community, found four isolates from dogs (23). Two of the isolates were resistant to several antibiotics, and they were assigned to ST25, ST250, and ST753. Recently, including both animal (cats, dogs, and horses) and human isolates from France, Lupo et al. found that some lineages within ST25 were composed of closely related isolates of animal and human origin (25). Thus, a relevant trend is that in the case of companion animals, some isolates recovered belonged to well-known human ICs; in particular, IC1, IC2, IC3, and IC7 (see Table 1). This implies that, to some degree, humans and their pets share similar STs. *A. baumannii* has also been described in livestock and wildlife. A study in Lebanon sampling fecal specimens from cattle, fowl, and pigs over 4 months in 2013 found five *A*. *baumannii* isolates (26). Those isolates belong to ST2, ST20, ST491, ST492, and ST493 and were resistant to most antibiotics, including carbapenems (26). Of note, ST2 and ST492 belong to IC2, whereas ST20 belongs to IC1; these two ICs are among the most important ICs in humans. Another study reported a 2017 chicken isolate (ABF9692) from a poultry farm in China (27). The isolate was pan-resistant and belonged to ST23 (Pasteur MLST scheme), which is part of IC8. A study looking for *A. baumannii* in turkeys farmed for meat production found that, although this bacterial species had a low prevalence, the isolates found were considerably diverse (18 different STs, Oxford MLST) and susceptible to many antibiotics (28). Of note, *A. baumannii* has also been reported in other farmed animals, such as mink (29). Another study taking a One Health stance analyzed 15 cattle and pig isolates from Scotland in the context of the main human ICs and noted that the Scottish isolates formed novel lineages (clones) not related to the human ICs (9). Importantly, these Scottish isolates had fewer ARGs than the human isolates. Therefore, in this case, humans, cattle, and pigs do not share similar STs. A study that sampled poultry (chicken and geese) but also white stork (a wild bird) found that neither the poultry isolates nor the white stork isolates were genetically differentiated from the human samples (30), demonstrating that humans, poultry, and white storks have similar STs. Remarkably, *A. baumannii* has also been isolated from the gut and gills of *Pagellus acarne* fish off the Algerian coast (31). These isolates belonged to ST2, a member of IC2. Wilharm and colleagues conducted the most extensive study to date, spanning time, geography, and sample size, analyzing over 1,000 isolates from white storks, soil, earthworms, and other sources (13). Remarkably, analyzing the genetic diversity of the intrinsic Oxa-Ab (OXA-51-like) β-lactamase, they noted that the isolates collected had more than 50% of the known diversity of the human clones (13). Therefore, another important trend is that *A. baumannii* found in livestock and wildlife can be novel lineages, but in other instances belong to well-known human ICs, such as IC2 or IC8 (see Table 1).

*A. baumannii* has been found in different types of food (32). Raw and undercooked meat is an important cause of foodborne diseases worldwide, and this bacterium has been found in meat from different types of animals (7, 33–35). In one of the first studies using MLST to genotype raw meat isolates (beef, pork, poultry, and veal), Lupo et al. studied the prevalence of *A. baumannii* in raw meat sold in Switzerland (7). The authors found *A. baumannii* in 25% (62 out of 248) of the samples, where poultry was the meat with the major incidence (48%) of *A. baumannii*. Furthermore, a high diversity of genotypes (with 29 different STs, 24 of them novel) was found. Another study analyzing isolates sampled from a market in Lima, Peru, found 12 isolates belonging to several *Acinetobacter* species (35). The only *A. baumannii* isolate was assigned to ST273 (35), which was an ST also found in the study by Lupo et al. (7). Considering isolates from different types of meat (bovine, camel, chicken, caprine, and turkey) collected from butchers in the province of Isfahan in Iran, it was found that 20.10% (39 out of 194) of samples had *A. baumannii* (33). Ovine meat had the highest prevalence with 32.14%, while turkey meat had the lowest prevalence with 11.11%. In one of the most recent studies, Hazme et al. collected 70 *A*. *baumannii* isolates from raw meat in France and Switzerland (34). The isolates came from beef, chicken, pork, turkey, and veal and were assigned to 49 STs. Although some of the STs found do not belong to the main human ICs, some of them do; for instance, several isolates belong to IC11 (34). Produce can also be an important cause of foodborne illness, and *A. baumannii* has been reported in produce several times (10, 36–39). A recent study in Eastern Spain focusing on fresh organic vegetables found that around 5% of the samples harbored *A. baumannii* (40). Another study in the United States found a low prevalence of *A. baumannii* in fresh vegetables from a farmers’ market, yet these few isolates were highly resistant to clinically relevant antibiotics (39). One of the main drawbacks of many studies analyzing produce is that they often lack detailed genotyping (i.e. ,MLST or genome sequencing) of the isolates. In this respect, a study analyzing produce from Jordan was the exception (36). The authors studied an array of fruits and vegetables, analyzing 234 samples in total and found that 6.5% of the vegetable samples and 8.5% of the fruit samples were positive for the bacterium. They were also able to show that some isolates belong to STs previously described in clinical (human) isolates, yet some others belong to novel STs not previously described (36). Taken together, the above studies show that fruits and vegetables, in addition to meat, can be reservoirs of *A. baumannii*. Also, as with animals, both novel and human-associated lineages can be found in food products. In this regard, a very relevant aspect in terms of public health is the risk that *A. baumannii* in food products poses to (vulnerable) human patients. Importantly, food products can act as a shuttle, introducing *A. baumannii* into hospitals and nursing homes and other settings where vulnerable people dwell.

**TABLE 2:** Data available for *A. baumannii* from different sources

Classification	Sources	Data on molecular epidemiology	Data on AMR	Data on virulence
Human	Human	Substantial	Substantial	Considerable
Animals	Companion animals, livestock, wildlife	Some	Some	Little
Food	Meat, produce, dairy products, etc.	Some	Some	Very little
Aquatic	Rivers, lakes, WWTP	Some	Some	Very little
Soil	Soil	Little	Little	No data
Plants	Plants	Little	Little	No data

### *A. baumannii* is found in different aquatic environments

Several studies have shown that *A. baumannii* can dwell in aquatic environments (11, 12, 41–43). Conducting a systematic tracking of *A. baumannii* at the different stages of a Waste Water Treatment Plant (WWTP) for a year in Zagreb, Croatia, a study found that 86% of the 119 *A*. *baumannii* isolates found were carbapenem-resistant (43). Importantly, of the 16 isolates chosen for WGS, 10 isolates belonged to ST2 and thus IC2 (Pasteur scheme), 2 were ST1 (ergo, IC1), 1 was ST79 (IC5), and 3 did not belong to any known ICs. The high abundance of IC2 in these WWTP isolates might reflect the prevalence in hospital settings in the local area. A recent study in Nigeria analyzing the presence of carbapenem-resistant *A. baumannii* in hospital wastewater over a year found 77 *A*. *baumannii* isolates (44). These showed high genetic diversity, as most of them belong to novel STs. Only a few isolates were assigned to STs from IC1 (nine isolates), IC8 (five isolates), and IC9 (one isolate). These 77 isolates had high resistance rates, having *bla_NDM-1_* and *bla_OXA-23_* as the most common carbapenem resistance genes. These studies show that *A. baumannii* can be found in WWTPs and that the release of improperly treated water into the environment can contribute to the dissemination of (resistant) *A. baumannii*. Sampling different cities and suburbs in South Africa (comprizing mostly WWTP isolates), a study found that all but one of the isolates belonged to the novel ST2520, and one isolate was assigned to IC1 (42). These environmental isolates showed significantly more susceptibility to the antibiotics tested than the clinical isolates (42). One recent study went far beyond one source and explored different aquatic environments: agricultural water, urban streams, effluents from WWTP, and even tank milk (11). Importantly, some of the isolates from these aquatic sources were novel STs, well differentiated from the ICs, highlighting the unexplored diversity within the environmental isolates of *A. baumannii*. Another recent study conducted a genomic analysis of 10 *Acinetobacter* isolates sampled from aquatic sources in South Australia in 2019 (12). The isolates belong to six different known *Acinetobacter* species, with one isolate being *A. baumannii*, which was recovered from a lake and was not closely related to the human ICs. The isolate was assigned to ST350, which had only been found in food in Switzerland (12). This implies that non-human lineages (STs) can be found in diverse non-human sources and distant geographic areas. *A. baumannii* has also been found in rivers (11, 45, 46). For instance, in 2010, an isolate belonging to IC2 (ST2) was recovered from the Seine (45). *A. baumannii* has also been described in water from an artisanal well in Koura in Lebanon (10); the isolate recovered was assigned to ST1, which falls within IC1, a clone frequently found in humans. *A. baumannii* has also been found in soil and grass (8, 46). Whereas the soil isolates belong to ST646, the grass isolates form highly diverged groups from one another, but were also not closely related to the main ICs (8). The major trends from these studies are: (i) that *A. baumannii* can be found in diverse aquatic environments, from WWTP to agricultural water, and even lakes and rivers; (ii) some of these isolates are closely related to human ICs, yet others are well diverged from human ICs.

**Fig 1:**
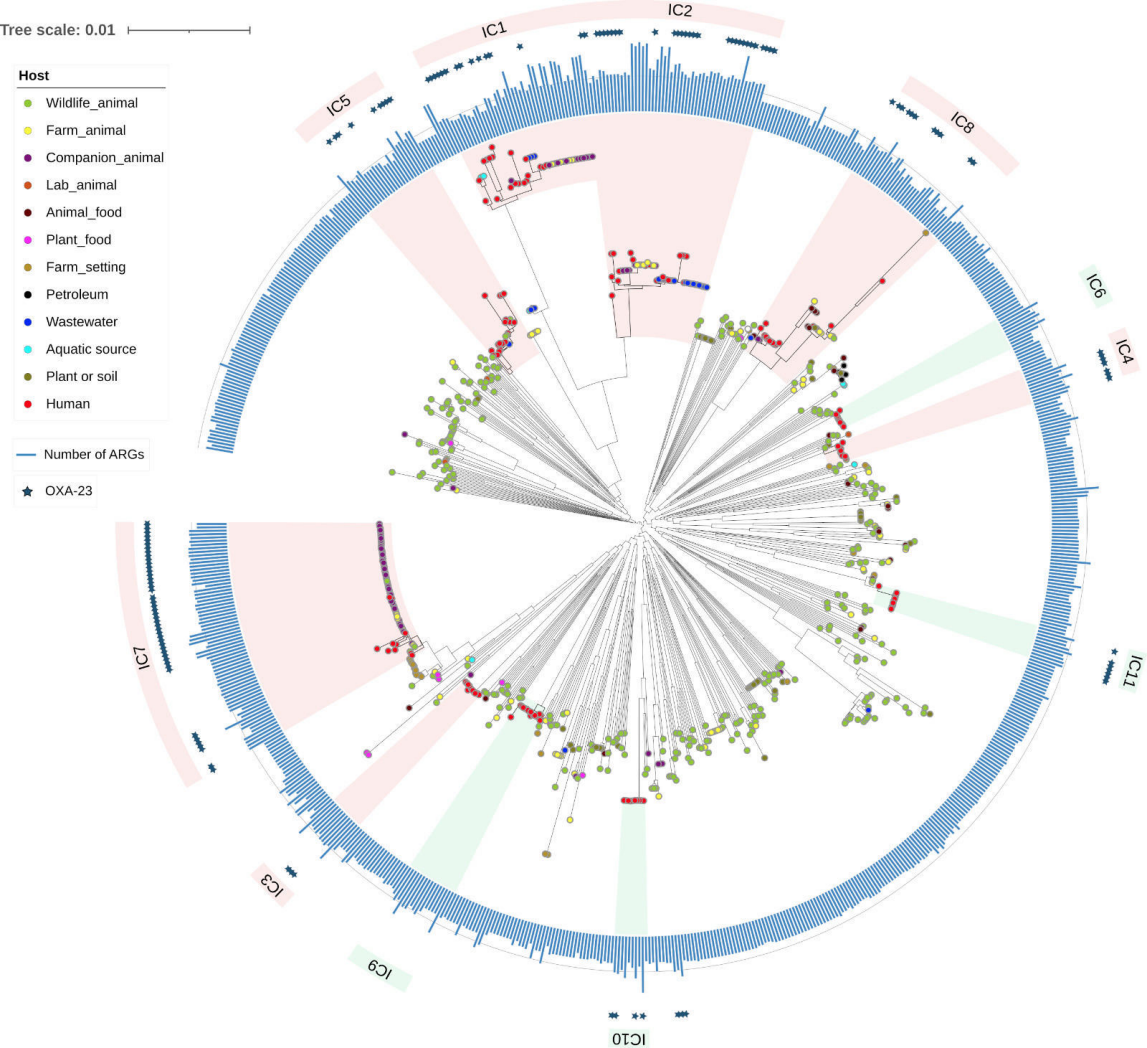
Core genome phylogeny of human and non-human isolates of *A. baumannii*. Phylogeny showing the phylogenetic relationships between human and non-human isolates. Colored dots denote the different hosts, see color key. The external circle gives the different ICs and the bars next to the strain names show the number of antibiotic resistance genes (ARGs) per isolate. The salmon shades for the ICs mean that these ICs contain non-human isolates, whereas the green shades indicate ICs containing only human isolates. The presence of OXA-23 is denoted with stars. The scale bar shows substitutions per site. The line gives the median number of ARGs, which was 27.

**Fig 2:**
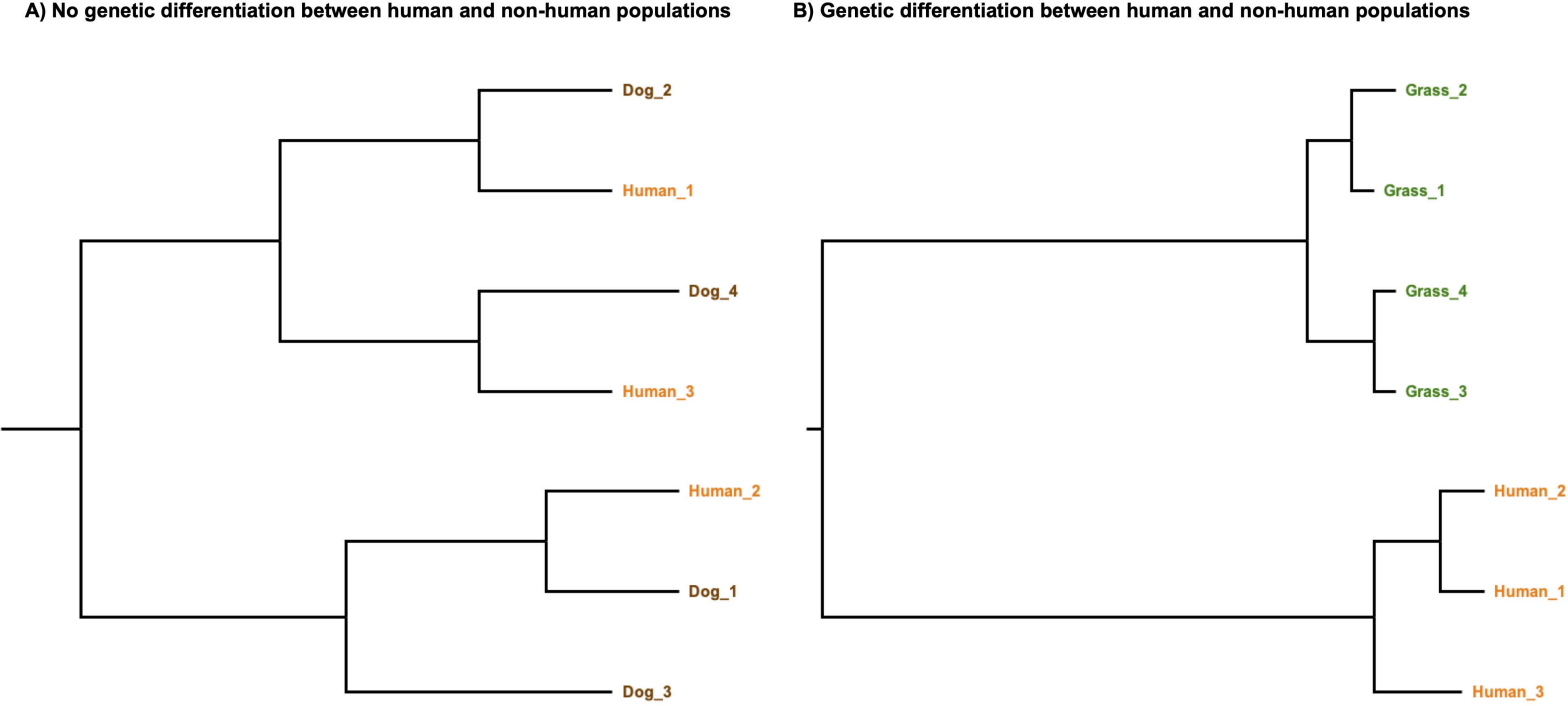
Scenarios of the relationships between human and non-human populations of *A. baumannii*. In the first scenario (**A**), human populations are not differentiated from non-human populations. Under a second scenario (**B**), human and non-human populations are well differentiated from each other. Colors denote different hosts: brown dogs, green grass, and orange humans. The branch lengths in both phylogenies represent genetic distance.

To better show the genetic relationships between the non-human and human population of *A. baumannii*, we constructed a core genome phylogeny of non-human and human isolates as we have done before (47–49). In our selection of the human ICs, we included two recently described ICs, namely IC10 and IC11 (50, 51). Table S1 provides a list of all the genomes employed for the phylogenetic analysis. The phylogeny (Fig. 1) shows that non-human isolates are dispersed all over the tree and are not confined to just a few clades. Furthermore, the tree shows that many non-human isolates (non-red dots) fall within human ICs (scenario 1); relevant cases are IC1, IC2, IC7, and IC8. In this first scenario, human and non-human populations are not genetically different from each other and have constant exchange between them. An example of this is ST25 (Pasteur MLST scheme) (25). Yet, the phylogeny also shows that some non-human isolates form lineages not closely related to the ICs (scenario 2). In this second scenario, human populations are genomically well-differentiated from non-human populations, and there is no exchange between them. Examples of these are recently described novel clones in cattle, pigs, and grass (8, 9). Figure 2 provides a schematic diagram of these two scenarios.

## ANTIBIOTIC RESISTANCE OF NON-HUMAN *A. baumannii*

*A. baumannii* has a very dynamic resistome (52). The bacterium is intrinsically resistant to a large number of antibiotics due to the presence of chromosomally encoded β-lactamases and efflux pumps, and its ability to downregulate porin expression (53). More recently, some members of the species have become proficient at acquiring ARGs from exogenous sources. These strains demonstrate a high degree of plasticity, in particular due to a large and varied repertoire of mobile elements such as plasmids, transposons, insertion sequences (3), and XerCD-*dif* sites (54), aided by the naturally transformable nature of the species (55). These features combined have contributed to the success of *A. baumannii* as an opportunistic human pathogen. Isolates taken from clinical sources are now likely to be highly drug-resistant, and in many areas of the world (particularly in low and middle-income countries), the isolates can be resistant to all available conventional treatment options. In contrast, *A. baumannii* from non-human sources can vary hugely in their antibiotic resistance phenotype and genotype.

### Resistance in companion animals

Probably the largest number of published studies into non-human *A. baumannii* have focused on companion animals. Among these, most isolates have come from cats and dogs, with a minority of isolates from a range of other animals or veterinary clinic environments. Typically, bacteria isolated from companion animals are reported as being MDR, including to clinically relevant antibiotics. However, this varies largely depending on when the isolates were collected and, importantly, the sample sizes are small, making generalizable conclusions difficult (see Table S2). Earlier studies tended to have isolates that were susceptible to ciprofloxacin, piperacillin-tazobactam, aminoglycosides, and carbapenems (56–58). In some cases, isolates had raised carbapenem MICs largely due to IS*Aba1* insertion upstream of the *oxaAb* gene (57), but acquired resistance genes were uncommon (56). In a few isolated cases, there were reports of MDR *A. baumannii* carrying the *oxa23* carbapenemase, but these were rare (20, 57, 59).

More recent studies have reported that companion animal isolates tend to be MDR, including resistance to ciprofloxacin, piperacillin-tazobactam, aminoglycosides, and carbapenems, and to carry acquired resistance genes such as *oxa23* and *bla*_NDM-1_ carbapenemases (25, 60–62). It is notable that amongst these isolates, those sampled from diseased animals largely belonged to recognized ICs (e.g., IC2 and IC7) and were more antibiotic resistant, whereas those isolated from the veterinary environment were more susceptible and belonged to non-clonal MLST sequence types (25, 60, 61). These patterns in phenotypic susceptibility and antibiotic resistance genotype mirror the epidemiology of clinical *A. baumannii*, including similar plasmids and genetic structures such as transposons carrying ARGs (25, 60, 61), suggesting that human clinical isolates and companion animal isolates share a common history.

### Resistance in livestock and food

There has been interest in determining whether *A. baumannii* exists within the food chain and whether this could be a source of resistant strains that could subsequently cause infection. The level of antibiotic resistance and resistance gene complement of food and food animals varies between the limited number of studies in this area, and the variety of sample types used (fecal material, swabs of live animals, carcasses, raw meat, fresh vegetables, and fruit) makes it difficult for firm conclusions to be drawn.

#### Livestock

Antibiotic-susceptible *A. baumannii* have been isolated at low frequencies from carcasses (9), and in a sample of 1,293 carcasses from China only a single *A. baumannii* was isolated, though this was reported as an MDR strain carrying *bla*_NDM-1_ (63). When live animals have been screened, isolated *A. baumannii* are largely susceptible to clinically relevant antibiotics and lack acquired resistance genes (10, 30). *A. baumannii* can be more frequently isolated from fecal samples, but in most cases, the strains were susceptible to antibiotics even when they belonged to established ICs (10, 28, 64). An exception to this was a small study in Lebanon that isolated five strains from a cow, a pig, and birds. All were resistant to carbapenems and carried *oxa23*, with one strain also carrying *oxa58*, but all were unique MLST sequence types, suggesting a possible spread of the resistance genes across genotypes, though the study does not state whether the isolates all came from the same farm or not (26). However, given that a carbapenem was employed as part of the screening (thus selecting for carbapenem-resistant phenotypes), it is not possible to tell if these animals mainly carried carbapenem-resistant strains.

#### Raw meat

Raw meat has been identified as a source of *A. baumannii*, but the levels of antibiotic resistance vary. Two studies sampling meat from two different provinces in Iran found isolates that were resistant to carbapenems and aminoglycosides and identified various β-lactamases including *oxa23*, *oxa40*, *oxa58*, *bla_VIM_*, *bla_IMP_*, and *bla_SIM_* (33, 65). In contrast, a study of raw meat from Switzerland found that while *A. baumannii* could be isolated frequently, particularly from poultry, the strains were susceptible to clinically relevant antibiotics (7). Of note, this reflects the differences in resistance in clinical isolates in these two countries, where highly drug-resistant isolates are very common in one country but rare in another.

#### Fruit and vegetables

Few studies have assessed fruits and vegetables for their carriage of *A. baumannii*, and they have found contrasting results. Strawberries in Egypt carried *A. baumannii* that were susceptible to clinically relevant drugs (66), while potato and lettuce in the USA carried imipenem-resistant strains (39). In Spain, organic leafy vegetables and strawberries were found to harbor carbapenem-resistant isolates that carried *bla*_VIM_, *oxa48,* and *bla*_KPC_ genes, of which the latter two are unusual in *A. baumannii* (40). This study also found Enterobacteriaceae and non-fermenting Gram-negative bacteria in these samples, suggesting that resistance genes may be being transferred between genera in this environment.

### Resistance in wildlife and the natural environment

There is a distinction between antibiotic resistance in *A. baumannii* strains that are isolated from what might be considered human-contaminated environmental sources, and those that are from less contaminated sources.

#### Contaminated environments

Isolates from wastewater treatment plants, or water samples taken downstream of these plants, typically share similar characteristics with clinical isolates and are resistant to carbapenems and aminoglycosides, and carry *oxa23*, occasionally *oxa40(72*), and a range of aminoglycoside resistance genes (11, 41, 43). Interestingly, isolates obtained from the watercourse upstream of these plants are typically antibiotic-susceptible and don’t carry clinically relevant resistance mechanisms (34). Along these lines, the isolate from the Seine River was resistant to beta-lactams (carbapenems included), chloramphenicol, fluoroquinolones and quinolones, tetracycline, and tigecycline (45).

#### Uncontaminated environments

While it may be rare to find environments that have not been contaminated to some degree by human activity, where bacteria have been isolated from less obviously human-contaminated sources, the majority of isolates tend to be susceptible to antibiotics and don’t carry acquired ARGs or the insertion sequences associated with them (11, 13, 42). White storks have been identified as a species that appears to be particularly prone to carrying *A. baumannii*. This seems to be specific to the species, as bacteria were not recovered from Black Storks (13). However, these animals do not seem to be a reservoir of ARGs as the isolated strains did not carry clinically relevant resistant genes, insertion sequences, or interrupted *comM* genes. Therefore, current evidence suggests environmental sources are only likely to harbor *A. baumannii* carrying clinically relevant antibiotic resistance if they have been recently contaminated by humans, in particular with wastewater.

Figure 1 shows an *in silico* prediction of the resistome (blue bars by the colored dots) for the genomes considered for the phylogenetic analysis. Consistent with trends noted in this section, non-human isolates (non-red dots) seem to have fewer ARGs, and just a few have *oxa23* (blue stars). However, a few non-human isolates can have as many ARGs as the human isolates.

## VIRULENCE POTENTIAL OF NON-HUMAN *A. baumannii*

Unfortunately, the virulence potential of *A. baumannii* from non-human populations has not received as much attention as the molecular epidemiology or antibiotic resistance aspects (see Table 2). *A. baumannii* possesses a range of virulence factors, reviewed extensively elsewhere (67–69), that contribute to its pathogenicity in hospital, community, agricultural, and environmental settings. Some noteworthy mechanisms of virulence are as follows:

### Adherence and biofilm formation

The ability to adhere to surfaces (70) and form biofilms (71, 72) enhances bacterial persistence in both environmental and host settings. It is conceivable that biofilms protect *A. baumannii* from various kinds of environmental stress and contribute to its persistence in the environment. Likewise, the outer membrane protein A (OmpA), the most abundant *A. baumannii* outer membrane protein (73), aids in the interaction of *A. baumannii* with eukaryotic cells, inducing cytotoxicity. OmpA has been shown to be present in the environmental isolates of *A. baumannii* (11). Whether its role in environmental isolates mimics that observed in clinical isolates remains to be seen.

### Capsular polysaccharide

Capsular polysaccharides have been considered a major virulence factor in *A. baumannii* (74) and have been described to be important in the virulence of clinical isolates globally (75–77). A recent study (11) showed that the production of capsules in non-human isolates is common. However, it remains to be seen if and how the production of capsules contributes to the virulence potential of environmental isolates.

### Lipooligosaccharide (LOS)

*A. baumannii* does not contain lipopolysaccharide (LPS) since it does not produce the O-antigen; instead, its outer membrane consists of LOS (69). Lipid A, a component of both LPS and LOS, is immunostimulatory and thus an important component of the virulence mechanism of various Gram-negative species, including *A. baumannii*. The study by Sykes et al. (11) showed a degree of conservation of *lpxA* and *lpxC*, LOS biosynthetic genes, in the non-human isolates of *A. baumannii*, suggesting the conservation of lipid A biosynthetic pathway in these isolates. The presence of the lipid A biosynthesis pathway is likely to contribute to the virulence potential of these isolates. However, studies investigating the role of lipid A in the virulence of such isolates are currently lacking.

### Iron acquisition systems

*A. baumannii* has developed efficient iron acquisition mechanisms (78), allowing it to thrive in iron-limited environments such as the human body. This capability is essential for bacterial growth and virulence. The prevalence of these mechanisms in the non-hospital isolates is poorly understood.

### Secretion systems

*A. baumannii* possesses a variety of secretion systems (79). The Type I secretion system (T1SS) is involved in the delivery of protein from the cytoplasm to the external environment of the bacterial cell (80). The T1SS has been shown to be present in the pathogenic strains of *A. baumannii* (80). The Type IV secretion system (T4SS) is involved in horizontal gene transfer, contributing to the mobilization of DNA and plasmids. The T4SS was recently shown to be widely prevalent among the *Acinetobacter* genus, including *A. baumannii* (81). Considering the role T4SS plays in the genetic transfer and the propensity of *A. baumannii* to horizontal gene transfer, this system is likely to be an important virulence factor in non-hospital isolates of *A. baumannii*. However, more work is needed to determine its prevalence in such isolates.

The Type VI secretion system (T6SS) has been shown to provide a competitive advantage to *A. baumannii* against other organisms (82), thus aiding in the colonization of *A. baumannii* in patients (83, 84). While our understanding of the prevalence of T6SS in non-hospital isolates remains poor, the presence of such a system is likely to lend a competitive advantage to *A. baumannii* in environmental settings.

Not surprisingly, there are only a few studies that have investigated the virulence of non-human isolates, and they show contrasting results. On the one hand, some isolates from different sources seem not to be as virulent as the human isolates. For instance, an isolate (NCIMB8209) recovered from the aerobic decomposition of guayule (a perennial woody shrub) had reduced virulence when compared with human isolates as observed in the *Galleria mellonella* and *Caenorhabditis elegans* infection models (85). Also, some animal isolates could have fewer virulence factors than human isolates. For example, conducting an *in silico* analysis of virulence-related genes, a study on pig and cattle isolates (9) found that animal isolates had fewer capsule genes, and some lacked genes for heme utilization. The study analyzing aquatic isolates from South Africa noted that these did not have the same ability to form biofilms as the clinical isolates (42). On the other hand, some isolates from different sources seem to be as virulent as the human isolates. To compare the virulence potential of avian isolates to that of human isolates, a study used the *G. mellonella* insect model and found that there was no significant difference between the LD_50_ doses of human clinical isolates and the avian isolates (30). They also evaluated adhesion to human lung epithelial cells and found that a couple of avian isolates were significantly better at adhering to these cells (30). Another study conducted an array of phenotypic assays and showed that environmental isolates from diverse sources (river, soil, mills, etc.) were serum-resistant, able to form capsules, and even able to kill *G. mellonella* (46). It has also been noted that the isolate SAAb472, which was sampled from a lake in Australia, had capsular polysaccharide and other virulence-related genes similar to those in clinical isolates (12). One of the most extensive studies in terms of different aquatic sources of *A. baumannii* found that virulence (as assayed by the *G. mellonella* model and *in silico* prediction of the virulome) was not associated with the source of origin, (human) clinical versus non-human, nor with geography (11). Notably, that study found some environmental isolates can kill *G. mellonella* as efficiently as clinical strains. Thus, it seems that non-human isolates, in some instances, can be as virulent as human clinical isolates; whereas on other occasions, they are not as virulent as the human isolates. Nevertheless, genetic studies show that non-human isolates appear to contain several virulence genes that have been reported in the human clinical isolates, and thus, one of the future research avenues to be pursued is to identify the specific mechanisms that might underlie virulence in non-human hosts.

## FINAL CONSIDERATIONS

Many studies have demonstrated that *A. baumannii* is more than a human pathogen. It is now considered a relevant pathogen in veterinary medicine (14, 15). However, further studies extensively sampling non-human sources are needed to establish the impact that non-human hosts/sources have in spreading *A. baumannii* in humans. Notably, some non-human sources might be more prone to be involved. For instance, companion animals, in very close contact with humans, are bound to be potentially important players. However, reversing the anthropogenic point of view and in a clear One Health angle, we could consider that humans can also be a reservoir of *A. baumannii* for other species. For instance, a study described a likely case of reverse zoonosis (zoonanthroponosis) as the source of an outbreak of pneumonia in sheep in Pakistan (86). Although there has been important progress considering the molecular epidemiology of non-human *A. baumannii*, there are still challenges to be addressed. First, although there have been studies about agricultural sources, hardly any attention has been paid to animals, plants, or environments not economically relevant for humans. Another very relevant challenge is the highly non-homogeneous sampling in terms of geography, as very few countries have sampled non-human *A. baumannii*. Furthermore, sampling is typically biased toward antibiotic-resistant isolates or isolates obtained from sick individuals. Considering this, there is evidence that some animals are colonized by *A. baumannii* without causing any apparent sickness (23, 24). An important aspect here is that in some instances, selective media was used, whereas in others not, thus complicating the comparisons of the different studies. Finally, most of what we know about *A. baumannii* is based on culture-dependent methodology. This fails to consider sources that are not trackable by microbial culture. In this regard, metagenome-assembled genomes (MAGs) are a promising avenue. Indeed, it was recently shown that some MAGs for *A. baumannii* came from atypical sources and represented novel diversity (87).

Considering the transmission between human and non-human populations, two scenarios are possible. One implies gene flow between human and non-human populations, which has been described in several STs from different ICs; outstanding cases are ST2 (IC2), ST23 (IC8), ST25 (IC7), and ST79 (IC5). In the other scenario, human populations are well differentiated from non-human populations, and there is no exchange between them, which is the case of the recently described clones from cattle, pigs, and grass (8, 9). The available data show that some lineages (ICs) are better equipped to inhabit different hosts/sources. Both IC1 and IC2 have been described in humans but also in companion animals, livestock, and even aquatic environments (10, 19, 20, 26, 43, 45). Also, in a given locale, there could be different lineages co-existing. For instance, the study of grass isolates found seven well-differentiated groups (8). This is similar to what has been reported in some hospitals (47, 48, 88), where multiple co-circulating lineages have been described . Importantly, considering the inaccuracy of the MLST schemes in *A. baumannii* (89), WGS should be the preferred option to determine the genetic identity of non-human populations under study. Also, few studies have put their findings in a proper global context (8, 9, 51, 90), which considers samples from the different human ICs.

Regarding antibiotic resistance, a few trends are evident. First, in general, non-human populations tend to have fewer ARGs, carrying mainly the intrinsic mechanisms such as efflux pumps or the *oxa51* β-lactamase. Second, the more the sources have been affected by or are in close contact with humans, the more similar the non-human isolates are to the clinical ones in terms of ARG content. For instance, isolates from WWTP have high levels of resistance. Of note, this was the case whether or not the isolates belonged to one of the main human ICs. Third, it is also important to note that extensively drug-resistant (XDR) and pandrug-resistant (PDR) phenotypes have already been described in isolates from non-human sources (27, 36). An important aspect for future studies is to determine which mobile genetic elements (MGEs) have a role in spreading antibiotic resistance in non-human populations and if these elements differ from those MGEs found in the human populations of *A. baumannii*. In this regard, some studies have suggested that non-human populations might have fewer plasmids and prophages than human ones (8, 91).

By far, the least studied aspect of the three themes has been the virulence of *A. baumannii* from non-human sources. Yet, the virulence capacity of these non-human populations could be an important factor in terms of public health (92). These non-human populations could be a possible source of infection for humans, making understanding their virulence a key concern (92). Remarkably, there is already data suggesting that, at least in some instances (11, 46), non-human isolates can be as virulent as human isolates. However, the limited number of existing studies makes it challenging to fully understand the virulence of non-human isolates. Since these isolates have been shown to possess several genes associated with *A. baumannii* virulence in clinical settings, researchers must move beyond genotyping and antibiotic resistance profiling to investigate their virulence potential more thoroughly. It is important to note that pathogenicity depends on the host-pathogen interactions, and these very likely will be different for different hosts.

*A. baumannii* is a One Health issue (17). Thus, it is necessary to analyze the non-human clones in the context of the molecular epidemiology of human clones. In this respect, discovering the reservoirs of *A. baumannii* is key if we are to fully characterize the transmission routes of this bacterium and, in turn, be able to curtail its spread. Hence, a global and multi-host sampling strategy is vitally important (93). Finally, other *Acinetobacter* species have also been found to inhabit different sources besides humans (94–96). Ergo, a One Health view would be desirable when analyzing the diversity of the whole genus.

## SUPPLEMENTAL MATERIAL

Table S1 aac.00801-25-s0001.xlsx List of all the non-human isolates plus representative genomes from the main ICs considered for the phylogenetic tree.

Table S2 aac.00801-25-s0002.docx Summary of the percentage of intermediate or resistant strains from *A. baumannii* isolated from companion animals.

ASM does not own the copyrights to Supplemental Material that may be linked to, or accessed through, an article. The authors have granted ASM a non-exclusive, world-wide license to publish the Supplemental Material files. Please contact the corresponding author directly for reuse.
