# Supplementary material for: Acinetobacter baumannii: much more than a human pathogen
Source: Antimicrob Agents Chemother. 2025 Jul 25;69(9):e00801-25. doi: 10.1128/aac.00801-25 (PMC12406671; doi:10.1128/aac.00801-25)
Supplement: Table S2 — Summary of the percentage of intermediate or resistant strains from A. baumannii isolated from companion animals. [file aac.00801-25-s0002.docx]

**Supplementary Table 2**

Summary of the percentage of intermediate or resistant strains from *A. baumannii* isolated from companion animals.

|  |  |  | **% I/R** | | | | | | | | | | |
| --- | --- | --- | --- | --- | --- | --- | --- | --- | --- | --- | --- | --- | --- |
| **Study** | **Year isolated** | **No. strains tested** | **AMK** | **CAZ** | **FEP** | **CIP** | **COL** | **GEN** | **IPM** | **MEM** | **TZP** | **SXT** | **TET** |
| Francey et al | 1995 - 1997 | 20 | 11 | 68 | - | 47 | - | 79 | 0 | - | - | 95 | 84 |
| Ewers et al | 2000 - 2013 | 25-61 | - | 100 | - | - | - | 89 | 5 | 5 | - | 51 | - |
| Endimiani et al | 2004 - 2009 | 19 | 0 | 16 | - | 63 | - | 89 | 16 | 16 | 32 | - | - |
| Naing et al | 2012 - 2014 | 7 | - | - | - | - | 0 | 86 | - | - | - | 86 | 86 |
| Lupo et al | 2010 - 2019 | 40-46 | 53 | 12 | 60 | 98 | - | 93 | 64 | 64 | 69 | - | - |
| Andre et al | 2021 - 2022 | 14 | 0 | 7 | 0 | 100 | 0 | 7 | 0 | 0 | 79 | 0 | 79 |

Only studies with more than one isolate were included. Importantly, these results very much depend on the screening method (i.e., with or without an antibiotic). Percentages are colour-coded with the lowest for that drug being dark blue and the highest being dark red. AMK, amikacin; CAZ, ceftazidime; FEP, cefepime; CIP, ciprofloxacin; COL, colistin; GEN, gentamicin; IPM, imipenem; MEM, meropenem; TZP, piperacillin-tazobactan; SXT, trimethoprim-sulfamethoxazole; TET, tetracycline.
